# Development of an MCL-1-related prognostic signature and inhibitors screening for glioblastoma

**DOI:** 10.3389/fphar.2023.1162540

**Published:** 2023-07-19

**Authors:** Ao Zhang, Zhen Guo, Jia-xin Ren, Hongyu Chen, Wenzhuo Yang, Yang Zhou, Lin Pan, Zhuopeng Chen, Fei Ren, Youqi Chen, Menghan Zhang, Fei Peng, Wanting Chen, Xinhui Wang, Zhiyun Zhang, Hui Wu

**Affiliations:** ^1^ Department of Neurology, The First Hospital of Jilin University, Changchun, China; ^2^ Department of Cardiology, The First Affiliated Hospital of Sun Yat-sen University, Guangzhou, China; ^3^ Department of Neurology, Stroke Center, The First Hospital of Jilin University, Changchun, China; ^4^ Department of Neurosurgery, State Key Laboratory of Oncology in South China, Collaborative Innovation Center for Cancer Medicine, Sun Yat-sen University Cancer Center, Guangzhou, China; ^5^ Clinical College, Jilin University, Changchun, China; ^6^ Department of Clinical Laboratory, The Fifth Affiliated Hospital of Xinxiang Medical College, Xinxiang, China; ^7^ Department of Medicine, Division of Endocrinology, Diabetes and Metabolism, Baylor College of Medicine, Houston, TX, United States; ^8^ Department of Hematology, The First Clinical Medical School of Lanzhou University, Lanzhou, Gansu, China; ^9^ Department of Plastic Surgery, The First Hospital of Jilin University, Changchun, China; ^10^ Department of Ophthalmology, First Hospital of Jilin University, Changchun, China

**Keywords:** glioblastoma (GBM), MCL-1, nomogram, virtual screening, molecular docking

## Abstract

**Introduction:** The effect of the conventional treatment methods of glioblastoma (GBM) is poor and the prognosis of patients is poor. The expression of MCL-1 in GBM is significantly increased, which shows a high application value in targeted therapy. In this study, we predicted the prognosis of glioblastoma patients, and therefore constructed MCL-1 related prognostic signature (MPS) and the development of MCL-1 small molecule inhibitors.

**Methods:** In this study, RNA-seq and clinical data of 168 GBM samples were obtained from the TCGA website, and immunological analysis, differential gene expression analysis and functional enrichment analysis were performed. Subsequently, MCL-1-associated prognostic signature (MPS) was constructed and validated by LASSO Cox analysis, and a nomogram was constructed to predict the prognosis of patients. Finally, the 17931 small molecules downloaded from the ZINC15 database were screened by LibDock, ADME, TOPKAT and CDOCKER modules and molecular dynamics simulation in Discovery Studio2019 software, and two safer and more effective small molecule inhibitors were finally selected.

**Results:** Immunological analysis showed immunosuppression in the MCL1_H group, and treatment with immune checkpoint inhibitors had a positive effect. Differential expression gene analysis identified 449 differentially expressed genes. Build and validate MPS using LASSO Cox analysis. Use the TSHR HIST3H2A, ARGE OSMR, ARHGEF25 build risk score, proved that low risk group of patients prognosis is better. Univariate and multivariate analysis proved that risk could be used as an independent predictor of patient prognosis. Construct a nomogram to predict the survival probability of patients at 1,2,3 years. Using a series of computer-aided techniques, two more reasonable lead compounds ZINC000013374322 and ZINC000001090002 were virtually selected. These compounds have potential inhibitory effects on MCL-1 and provide a basis for the design and further development of MCL-1 specific small molecule inhibitors.

**Discussion:** This study analyzed the effect of MCL-1 on the prognosis of glioblastoma patients from the perspective of immunology, constructed a new prognostic model to evaluate the survival rate of patients, and further screened 2 MCL-1 small molecule inhibitors, which provides new ideas for the treatment and prognosis of glioblastoma.

## Graphical Abstract

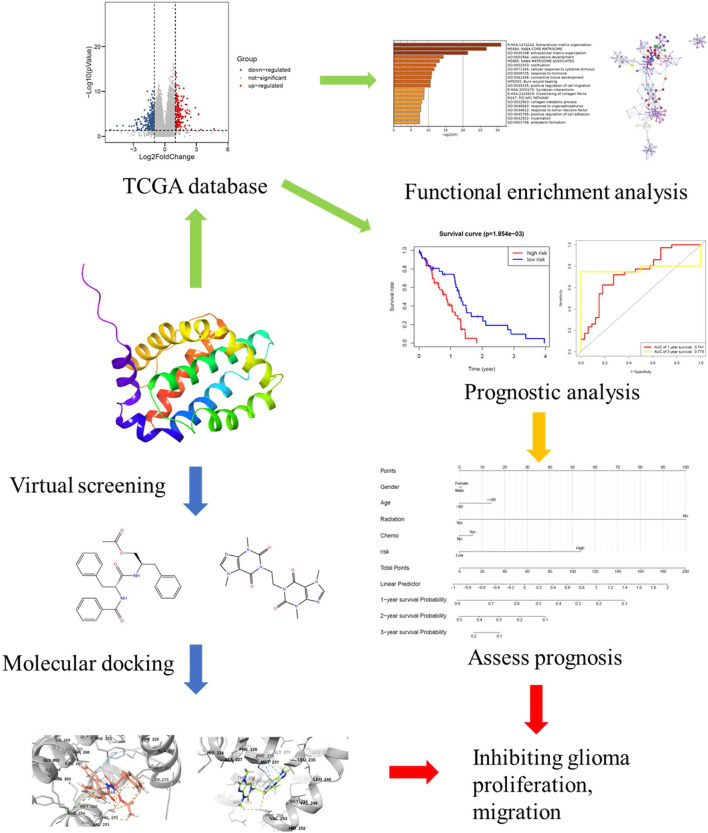

## 1 Introduction

Glioblastoma (GBM) is a brain tumor originating from glial progenitor cells and is the most common primary malignant tumor of the brain, accounting for 81% of malignant brain tumors (Ostrom et al., 2014; Xu et al., 2020; Li et al., 2023). Among them, glioblastoma has the highest and increasing incidence, but no curative treatment is available (Ostrom et al., 2019). The survival time of most patients is much lower than that of patients with other tumors, and the quality of life is very poor (Finch et al., 2021). At present, the conventional treatment methods for GBM include tumor resection, radiotherapy combined with temozolomide (TMZ) and targeted therapy with bevacizumab, etc., but these treatments have more or less obvious limitations (Allahyarzadeh Khiabani et al., 2023; Boongird et al., 2023; Hotchkiss et al., 2023; Jatyan et al., 2023; You et al., 2023). Therefore, how to predict the prognosis of GBM patients more accurately and intervene the factors affecting the prognosis, formulate more reasonable and effective treatment plans, and develop safer and more effective drugs are the key to treating glioblastoma patients.

Myeloid cell ischemia-1 (MCL-1), as a member of the B-cell lymphoma 2 (BCL-2) protein family, is one of the most frequently amplified genes in all human cancers including glioblastoma (Xiang et al., 2018). MCL-1 has three BH domains (BH1, BH2, and BH3), a C-terminal TM domain, and a large N-terminal region (Li S. et al., 2021). The four binding pockets (P1-P4) of MCL-1 interact with hydrophobic residues H1-H4 of only-BH3 protein, respectively, where the P2 and P3 pockets are the locations of “hot spot” residues for protein-protein interaction in MCL-1. This is different from anti-apoptotic proteins such as BCL2 (P4/P1 and P2) or BCL-XL (P2 and P4) and facilitates the design of specific MCL-1 inhibitors (Denis et al., 2020). Based on the structure of MCL-1, its role in apoptosis is promoting cell survival by interfering in the cascade of the events that cause trigger cell death and MOMP (Sancho et al., 2021). Numerous previous studies have demonstrated that MCL-1 is extremely important for glioblastoma, which can be used as a key target to inhibit the activity of glioblastoma cells. Downregulation of MCL-1 expression significantly induced apoptosis of tumor cells (Premkumar et al., 2013; Gratas et al., 2014; Jane et al., 2016; Juric et al., 2021). Therefore, there is great potential for the development of MCL-1 specific inhibitors for the treatment of glioblastoma, and it is crucial to create inhibitors that are both more efficient and less poisonous for the treatment of glioblastoma.

At present, the main strategies to design inhibitors against MCL-1 are based on the direct binding of BH3-mimetic to MCL-1, thereby releasing proapoptotic proteins and finally activating apoptosis (Li S. et al., 2021). Clinical studies for MCL-1 inhibitors have started for drugs such S64315, AZD5991, AMG 397, AMG 176, MIM1, etc. (Wei et al., 2020). These inhibitors have shown remarkable efficacy in the treatment of non-Hodgkin’s lymphoma, multiple myeloma, acute myeloid leukemia, B-cell lymphoma, and other hematological malignancies (Wei et al., 2020; Liu et al., 2021). However, in the treatment of glioblastoma, most MCL-1 small molecule inhibitors are not applicable due to the existence of blood-brain barrier. Among them, MIM1, as an identified BH3-mimetic, has promising biological and biophysical properties such as low molecular weight, ideal solubility, and stability. MIM1 reduced the viability of glioblastoma cells in a dose and time-dependent manner (Respondek et al., 2018). However, for other types of glioblastoma, the role of MIM1 has not been investigated so far.

The aim of this study is to establish an MCL-1-based prognostic model and to screen safe and effective MCL-1 inhibitors. Firstly, the RNA-seq and clinical data of 168 glioblastoma samples were downloaded from the TCGA database. According to the expression level of MCL-1, the samples were divided into MCL-1_L and MCL-1_H groups, and the enrichment levels of 29 immune signals in the two groups were analyzed. Functional enrichment analysis of these differentially expressed genes was performed. The MCL-1-associated prognostic signature was then constructed using lasso cox regression analysis. Finally, a nomogram prediction model was established to estimate the survival rate of glioblastoma patients. In addition, two small molecule inhibitors of MCL-1 were screened by a series of computer-aided techniques. With the development of drug research, natural products are playing an increasingly important role in molecular biology and drug exploration, which provide structural patterns for target compounds of new drugs and are an important source of new drugs. MIM1 was used as a reference drug in this study. NP (natural products) database in the ZINC database was virtually screened to explore potential MCL-1 inhibitors. Secondly, the pharmacological and toxicological characteristics of the compounds were analyzed. Molecular docking was then performed to assess the interaction between the selected compounds and MCL-1. The pharmacophore of the compound was also predicted. Therefore, a more suitable small-molecule MCL-1 inhibitor is required for the treatment of glioblastoma. Natural products are becoming a more significant part of molecular biology and drug discovery as drug research progresses since they offer structural patterns for target molecules of new medications and are a significant source of such pharmaceuticals. In this study, MIM1 served as the reference medication. Virtual screening was done on the natural products database in the ZINC database to look for probable MCL-1 inhibitors. Secondly, the pharmacological and toxicological properties of the compounds were examined. Then, molecular docking was used to evaluate how well the chosen drugs interacted with MCL-1. Additionally anticipated was the compound’s pharmacophore. Finally, using a molecular dynamics simulation, we examined the stability of the binding interaction. The study’s findings are summarized in a list of potential MCL-1 small molecule inhibitors and their pharmacological characteristics, which can support and assist the research on MCL-1 inhibitors and give further leads for the creation and advancement of glioblastoma therapy medications.

## 2 Materials and methods

### 2.1 Immunogenomic analysis, differential gene expression and functional enrichment analysis

RNA-seq and clinical data of 168 GBM samples were downloaded from TCGA (Cancer Genome Atlas database) website, and they were divided into 2 groups according to the expression level of MCL-1: MCL-1_L (*n* = 84) and MCL-1_H (*n* = 84). First, 29 immune signal enrichment levels were quantified in all glioblastoma samples, and single-sample gene set enrichment analysis (ssGSEA) score was used in this analysis. ssGSEA scores were used to analyze the activity or enrichment levels of different immune cell functions in each glioblastoma sample. All glioblastoma samples were then evaluated for the level of immune cell infiltration (immunoscore), stromal content (stromal score), and tumor purity. Finally, the expression of HLA genes and immune checkpoint genes in MCL-1_H and MCL-1_L groups was tested by ANOVA.

Rstudio and Wilcoxon Rank Sum And Signed Rank Tests were used to analyze the differential expression between MCL-1_H and MCL-1_L. Using |log2 fold change (FC)|>1 and adjusted *p* values < 0.05 as the cutoff criterion, all genes were analyzed. “limma” package was used for analysis, and 449 differentially expressed genes were obtained. Then “ggpubr” and “ggthemes” packages were used to visualize the expression levels of all genes, and the differentially expressed genes in the MCL1_H and MCL1_L groups were shown in volcano maps. The Metascape website (https://metascape.org) features gene annotation and visualization. The differentially expressed genes were uploaded to this website, and the gene ontology and signal pathway enrichment of these genes were analyzed.

### 2.2 Construction and validation of MCL-1-associated prognostic signature (MPS)

To construct an immune prognostic signature, we randomly divided the TCGA_GBM data set into two groups: training set and verification set. LASSO Cox analysis is a widely used high-dimensional predictive regressive method. By selecting the optimal penalty parameter lambda and using 10-fold cross-verification, shrinkage and variable identification can be achieved at the same time to prevent overfitting. To establish immune prognostic characteristics, we put DEGs into LASSO Cox regression and use the “glmnet” R package to proceed to analysis. By weighting Cox regression coefficients to estimate the risk score of each patient, MPS was created. Patients are classified as low-risk and high-risk “survivor” R packages obtained based on the best cut-off value of the risk score. The “survival ROC” R package was used to describe the receiver operating characteristic (ROC) curve to evaluate its sensitivity and specificity. Calculate the area under the curve (AUC) value according to the ROC curve. At the same time, the prognostic prediction ability of the MPS was further verified in the verification set.

### 2.3 Development of the nomogram

We assessed the independent prognostic power of MPS by univariate and multivariate Cox analyses. And based on the result of Cox analyses, we use the “rms” package to develop an innovative nomogram. Calibration charts for the probabilities of observing and predicting 1- year OS were performed to determine accuracy.

### 2.4 Virtual screening based on structure using Libdock, ADMT and TOPKAT

The Discovery Studio 2019 software, from BIOVIA in San Diego, California, United States, provides researchers with easy-to-use tools for protein simulation, modification, and precision medicine (Zhong et al., 2021; Zhong et al., 2022). In addition, we used the ZINC database, a free virtual screening database for commercial chemicals, as a ligand database. 3D molecular formats for 17,931 natural, named, and purchasable chemicals were initially obtained from the ZINC15 database. Both the 1.35 Å crystal structure of human MCL-1 (Protein Data Bank identifier: 6UDV) and the 3D structure of the positive reference medication MIM1 were imported into the LibDock working environment. To identify potential MCL-1 inhibitors, the ligand binding pocket domain of MCL-1 was selected as the binding site. The molecular docking between MIM1 and the treated MCL-1 was found to be successful. This site was therefore used as the active site for docking. All downloaded small molecule files were linked to this active site through the libdock module for preliminary virtual screening. All compounds’ docking postures were graded based on their LibDock score. The ADME module of Discovery Studio 2019 was used to calculate the absorption, distribution, metabolism and excretion (ADME) levels of the top 30 compounds, and the TOPKAT module was used to analyze the toxicological characteristics of the compounds. Finally, two molecules were selected as candidates.

### 2.5 Molecular docking and pharmacological analysis

Studies on molecular docking of ligands and proteins were conducted using the CDOCKER module of Discovery Studio 2019. Using the CHARMm force field, CDOCKER is a method that generates very accurate molecular docking statistics (Wu et al., 2021; Yang et al., 2022). During docking, the CDOCKER algorithm is based on a simulated annealing protocol where the receptor remains rigid and the ligand is allowed to bend and dock with protein residues within the binding site to find the most suitable binding mode (Li H. et al., 2021). The section within a 13 Å radius of the ligand’s geometric center is referred as the binding site spot. Therefore, according to the interaction energy analysis of CDOCKER, the most suitable compound was selected for the next study. In addition, the best binding pose of the selected compounds to the protein was demonstrated using the Schrodinger software.

### 2.6 Molecular dynamic simulation

The best binding conformation of the ligand-MCL-1 complex obtained from the previous molecular docking step was selected for molecular dynamics simulations. Using an explicit periodic boundary solvated water model, the ligand-receptor complex was contained in an orthogonal box and solvated. Sodium chloride was added to the solution with an ionic strength of 0.145 to mimic the physiological environment. The following simulation protocols were used for the system: 500 steps of steepest descent and conjugate gradient minimization; 5 ps-equilibration simulations in a normal pressure ensemble at a temperature of 300 K (slowly driven from an initial temperature of 50 K); and 50 ps-MD simulation (production module) at NPT (normal pressure and temperature) with a time step of 1 fs. Long-range electrostatics calculations were performed using the particle mesh Ewald (PME) technique, and all hydrogen-containing bonds were fixed using an adaptation of the linear constraint solver technique. The Discovery Studio 2019 analysis trajectory procedure was used to construct a trajectory for root-mean-square deviation (RMSD), potential energy, and structural parameters using the original complex configuration as a reference.

## 3 Results

### 3.1 Immunogenomic analysis between MCL-1_H and MCL-1_L

**FIGURE 1:**
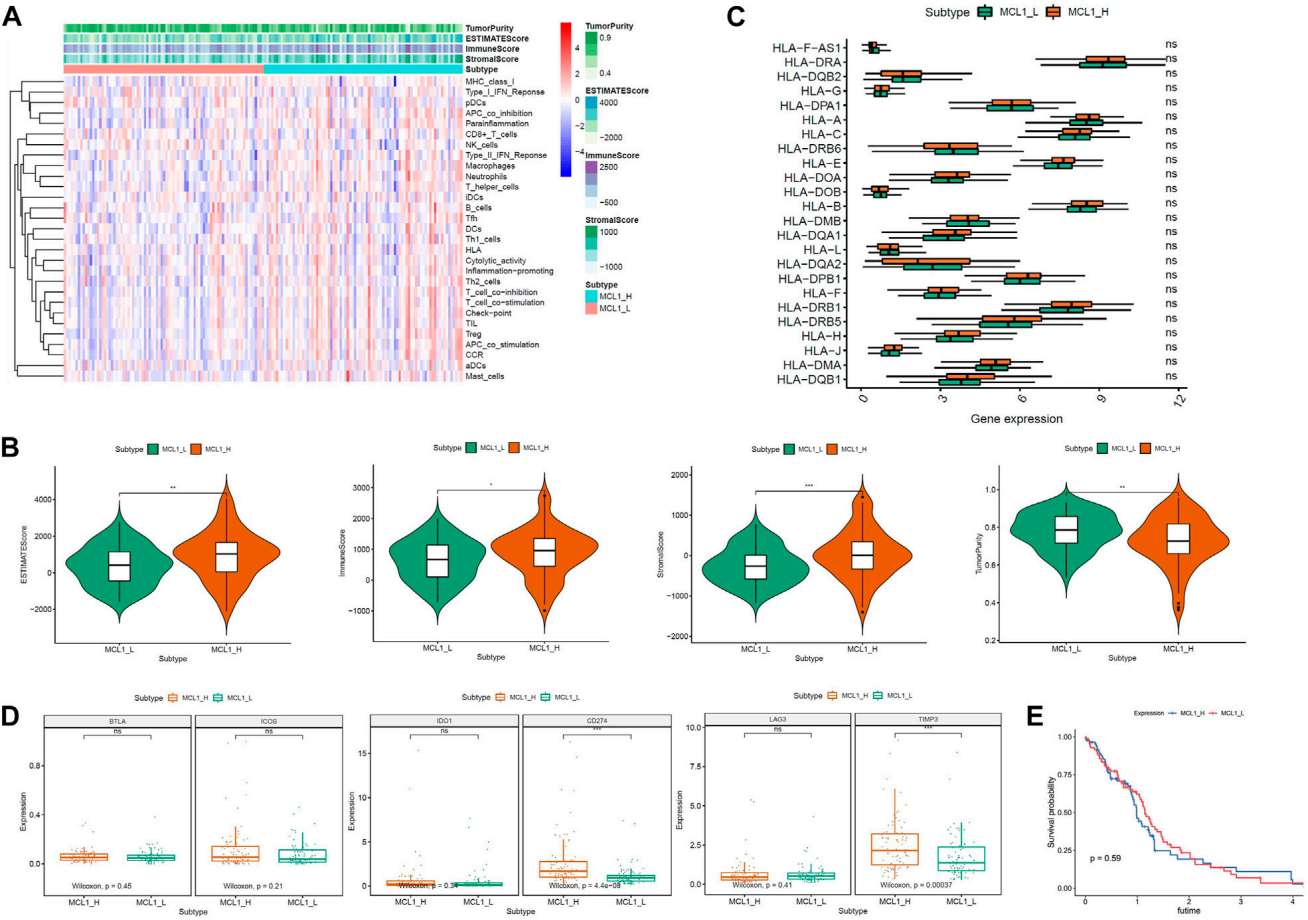
Immunogenomic analyses between MCL-1_H and MCL-1_L. **(A)** The enrichment levels of the 29-immune signature by ssGSEA score in each glioblastoma sample. ESTIMATE was used to evaluate Tumor purity, Stromal score and Immune score. **(B)** Comparison of the Immune score, Stromal score, ESTIMATE score, Tumor purity between MCL-1_H and MCL-1_L (Kruskal–Wallis test). **(C)** Comparison of the expression levels of HLA genes between MCL-1_H and MCL-1_L (ANOVA test). **(D)** Comparison of immune checkpoint gene expression levels between MCL-1_H and MCL-1_L (ANOVA test). **(E)** Survival curves of patients in MCL1_H group and MCL1_L group.

A total of 168 glioblastoma patients were included in this study. According to the expression level of MCL-1, they were divided into 2 groups: MCL-1_H (*n* = 84) and MCL-1_L (*n* = 84). Firstly, the expression levels of 29 groups of immune-related genes representing different immune cell types, functions, and pathways in glioblastoma samples were investigated. According to the ssGSEA score, we found that the enrichment levels of immune cells, functions, and pathways in the MCL-1_H and MCL-1_L groups were not significantly different (Figure 1A). The ESTIMATE results show that (Figure 1B), The MCL-1_H group had immune score (Kruskal–Wallis test, *p* < 0.001), stromal score (Kruskal–Wallis test, *p* < 0.001), and ESTIMATE score (Kruskal–Wallis test, *p* < 0.05) were higher than those of MCL-1_L group, but tumor purity (Kruskal–Wallis test, *p* < 0.001) was higher than that of MCL-1_H group. These results indicated that MCL-1_H contained more immune cells and stromal cells, and MCL-1_L contained more tumor cells.

The expression of HLA genes and immune checkpoint genes was next analyzed in the two groups. In the analysis of 24 HLA genes (ANOVA test, *p* < 0.05) (Figure 1C), the expression of 15 HLA genes was higher in MCL-1_H than in MCL-1_L. Including HLA-DRA, HLA-A, HLA-C, HLA-E, HLA-DOA, HLA-B, HLA-DQA1, HLA-L, HLA-DPB1, HLA-F, HLA-DRB1, HLA-H, HLA-J, HLA-DMA, HLA DQB1. Among the six immune checkpoint gene assays (Figure 1D), gene expression levels were higher in the MCL-1_H group than that in the MCL-1_L group, with significantly higher expression of CD274 and TIMP3 in the MCL-1-H group than in the MCL-1_L group. These results suggested that patients in the MCL-1_H group were immunosuppressed, and the use of immune checkpoint inhibitors, especially inhibitors of CD274 and TIMP3, had a positive effect on the treatment of MCL-1_H patients. In addition, when grouped according to MCL1 expression levels, there was no significant difference in prognosis between the two groups (*p* = 0.59) (Figure 1E).

### 3.2 Differentially expressed genes analysis

**FIGURE 2:**
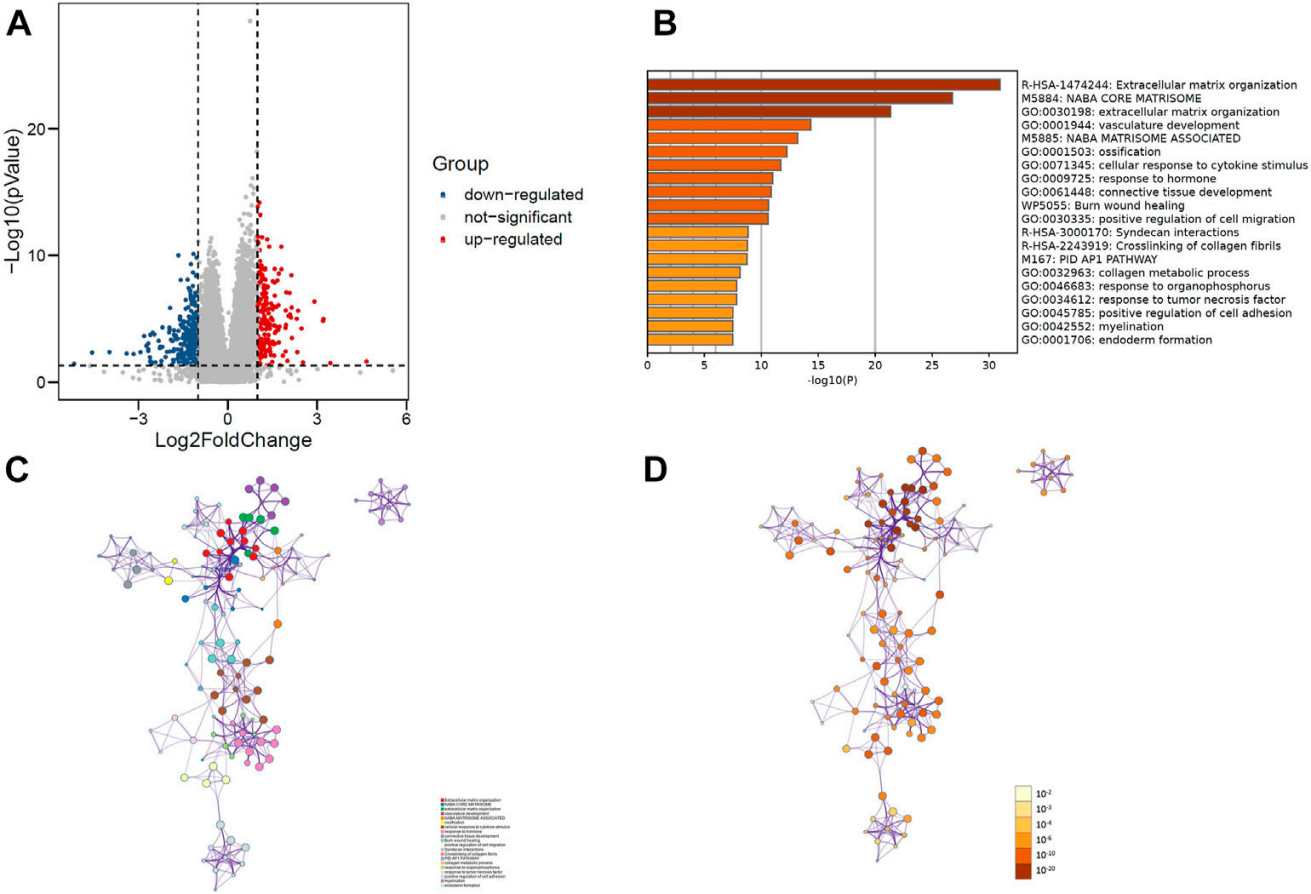
**(A)** Volcano plot of 449 genes differentially expressed between MCL-1_H and MCL-1_L. **(B)** Top 20 GO terms and KEGG pathways enrichment of DEGs. **(C)** Network plot colored by *p*-value, where terms containing more genes tend to have a more significant *p*-value in DEGs. **(D)** Network plot colored by cluster ID, where nodes that share the same cluster ID are typically close to each other in DEGs.

R software was used to analyze the information of 168 patients to determine the differentially expressed gene (DEG) data set. To FDR <0.05 and log2 fold change (FC) < 1 or more for the standard, there were 449 genes identified, of which 274 genes downregulated, 175 genes upregulated (Figure 2A). Importing 449 differentially expressed genes into the metascape website, it was found that these genes were mainly enriched in these terms: R-HAS-1474244: Extracellular matrix organization, M5884: NABA CODE MATRISOME, GO:0030198: extracellular matrix organization (Figures 2B–D). Where each node represents a cluster item and is colored by cluster ID and *p*-value.

### 3.3 The MCL-1 related prognostic signature (MPS) was constructed and validated

**FIGURE 3:**
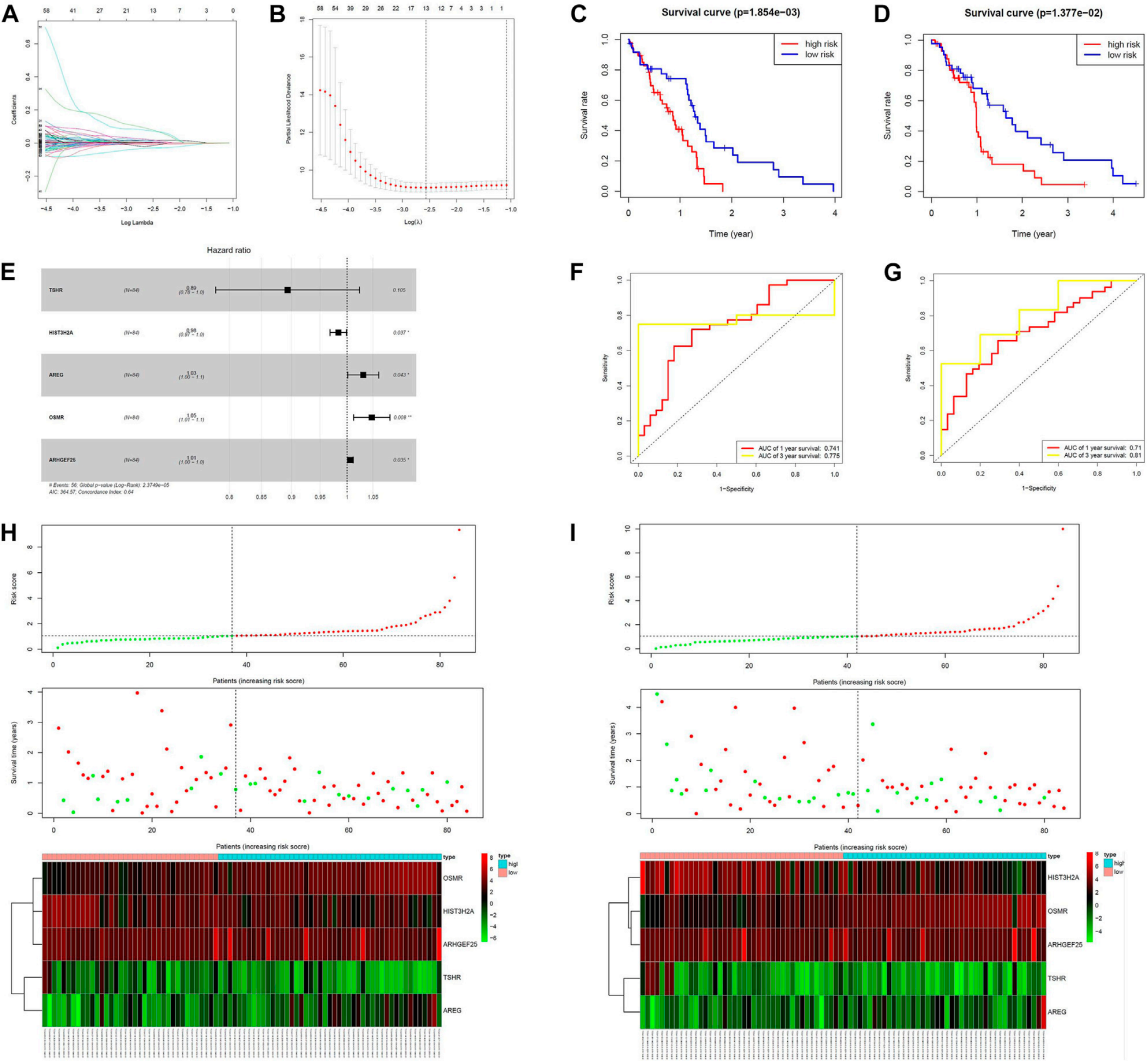
Construction of the MCL-1-related prognostic signature. **(A,B,E)** Kaplan–Meier curves of overall survival based on the MPS in the verification set and train set. **(C,D)** LASSO Cox analysis identified five genes most correlated to overall survival in the verification set and training set. **(F,G)** ROC curve analysis of the MPS. **(H,I)** Risk scores distribution, survival status of each patient, and heatmaps of prognostic five-gene signature in verification set and training set.

MPS were constructed in the training group using lasso cox regression analysis (Figures 3A, B, E) to calculate the risk score for each sample. The survminer package in R software was used to calculate the optimal cut-off value, and the patients in the training group were divided into 2 groups: high-risk group and low-risk group. The results of the Kaplan-Meier analysis indicated that the patients in the low-risk group had a better prognosis (Figure 3C). Figure 3H shows the distribution of risk scores [risk score = (−0.112721)*TSHR + (−0.016743)* HIST3H2A+ 0.030476*ARGE+ 0.046739*OSMR +0.005866*ARHGEF25] and gene expression in the training group. We analyzed the MPS of the training group by ROC curve, which showed high accuracy in predicting 1-year survival and 3-year survival (AUC of 1-year survival = 0.741, AUC of 3-year survival = 0.775) (Figure 3F).

We next validated the prognostic value of MPS using the same formula in the validation set. Similarly, all patients were divided into high-risk and low-risk groups. Figure 3D shows that patients in the high-risk group had a lower survival rate. Figure 3I shows the risk score and gene expression distribution of the validation group. Figure 3G demonstrates that MPS has high accuracy and sensitivity in predicting 1-year survival and 3-year survival (AUC of 1-year survival = 0.71, AUC of 3-year survival = 0.81).

### 3.4 Construct MPS-based nomogram model

**FIGURE 4:**
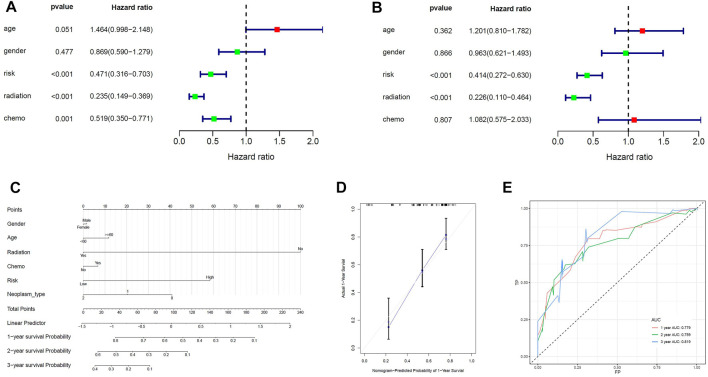
Construction of the nomogram model. **(A)** Univariate Cox analyses indicated that MPS was significantly associated with OS. **(B)** Multivariate Cox analyses indicated that MPS was significantly associated with OS. **(C)** Nomogram model for predicting the probability of 1- and 3-year OS in GBMs. **(D)** Calibration plots of the nomogram for predicting the probability of OS at 1 year. **(E)** ROC curve analysis of the nomogram.

Univariate cox analysis was first used to demonstrate that MPS was significantly associated with OS (Hazard ratio: 0.471%, 95% confidence interval: 0.316–0.703, *p* < 0.001), and then multivariate cox analysis was used to test the accuracy of MPS as an independent prognostic factor (Hazard ratio: 0.414%, 95% confidence interval: 0.272–0.630, *p* < 0.001) (Figures 4A, B). An MPS-based nomogram model was then developed (Figure 4C). A nomogram calibration plot predicting 1-year OS probability showed better agreement (Figure 4D). The AUC of 1-year, 2-year, and 3-year were 0.779, 0.759, and 0.819 (Figure 4E), respectively, which proved that the nomogram had good validity.

### 3.5 Virtual screening using Libdock, ADME, and TOPKAT of DS 2019

**FIGURE 5:**
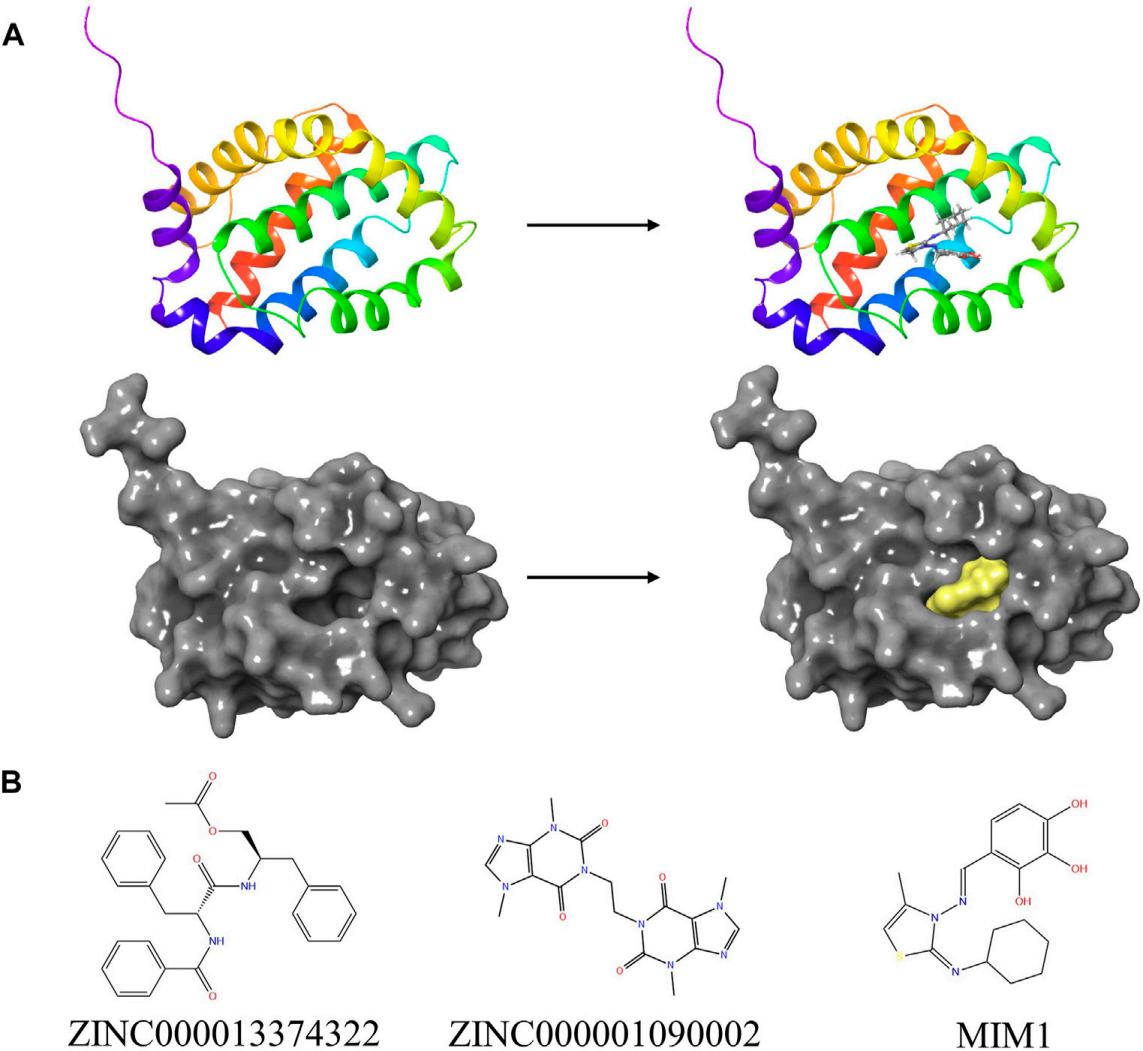
**(A)** The molecular structure of MCL-1 and the complex structure of MCL-1 with MIM1. Initial molecular structure was shown. The surface of the complex was added, yellow for MIM1 and gray for MCL-1. **(B)** The 2D structures of MIM1 and novel compounds were selected from virtual screening.

Based on the above results, MCL-1 proved to be a key target for the treatment of glioblastoma and influencing the prognosis of glioblastoma. Therefore, we used MCL-1 as a target for drug screening for the treatment of glioblastoma. The MIM1-MCL-1 complex binding pocket was an essential regulatory region that was chosen as a significant reference site for screening probable MCL-1 inhibitors. Figure 5A shows the 3D structures of MCL-1 (PDB ID: 6UDV) and the MIM1-MCL-1 compound. In accordance with the LibDock score, 4,854 compounds had a higher LibDock score than MIM1 (106.167). The top 30 compounds were chosen for additional investigation based on the LibDock score, and these 30 compounds are described in Table 1.

**TABLE 1:** Top 30 ranked compounds with higher LibDock scores.

Number	Compounds	Libdock score
1	ZINC000002572533	148.62
2	ZINC000040976869	147.033
3	ZINC000014883350	146.406
4	ZINC000100822245	145.294
5	ZINC000004096910	144.356
6	ZINC000230075702	144.266
7	ZINC000017044426	144.106
8	ZINC000004095521	143.012
9	ZINC000053147179	142.643
10	ZINC000003951623	142.538
11	ZINC000014951634	142.502
12	ZINC000049784088	141.387
13	ZINC000014883354	140.322
14	ZINC000013374322	140.085
15	ZINC000073280937	139.939
16	ZINC000014883346	139.835
17	ZINC000001577210	139.731
18	ZINC000019899011	139.665
19	ZINC000008552019	139.597
20	ZINC000038143593	138.888
21	ZINC000004104845	138.315
22	ZINC000014767590	138.235
23	ZINC000034944434	137.288
24	ZINC000002526388	137.022
25	ZINC000001090002	136.26
26	ZINC000008689960	136.136
27	ZINC000002097863	135.868
28	ZINC000005766341	135.342
29	ZINC000005811273	135.342
30	ZINC000006845904	135.298
	MIM1	106.167

Then ADME and TOPKAT modules of DS2019 were used to predict the pharmacological and toxicological properties of the top 30 compounds and MIM1. Based on the analysis in Tables 2, 3, ZINC000013374322 and ZINC000001090002 are not inhibitors of CYP2D6 and have a high intestinal absorption level. What’s more, these two compounds are weakly bound plasma proteins. More importantly, these two compounds showed no hepatotoxicity and no Ames mutagenicity. Figure 5B shows that these two compounds and MIM1 have similar six-membered and five-membered annular structures, reactive oxygen and nitrogen species atoms, suggesting that they may play similar roles. The compounds ZINC000013374322 and ZINC000001090002, which are expected to be promising candidates, were then investigated.

**TABLE 2:** Adsorption, distribution, metabolism, and excretion properties of compounds.

Number	Compounds	Solubility level	BBB level	CYP2D6	Hepatotoxicity	Absorption level	PPB level
1	ZINC000002572533	2	4	1	0	3	0
2	ZINC000040976869	3	4	0	0	2	1
3	ZINC000014883350	0	4	0	0	3	1
4	ZINC000100822245	2	4	1	0	3	0
5	ZINC000004096910	0	4	1	0	3	1
6	ZINC000230075702	2	4	1	0	2	0
7	ZINC000017044426	2	4	1	0	3	0
8	ZINC000004095521	0	4	0	0	3	1
9	ZINC000053147179	3	4	0	1	3	0
10	ZINC000003951623	2	2	1	1	0	1
11	ZINC000014951634	3	4	0	0	3	0
12	ZINC000049784088	4	4	0	0	3	0
13	ZINC000014883354	0	4	0	0	3	1
14	ZINC000013374322	2	2	0	0	0	0
15	ZINC000073280937	2	4	0	1	2	1
16	ZINC000014883346	0	4	0	0	3	1
17	ZINC000001577210	2	1	0	0	0	1
18	ZINC000019899011	2	2	0	1	0	1
19	ZINC000008552019	2	4	1	0	3	0
20	ZINC000038143593	3	4	0	0	3	0
21	ZINC000004104845	2	3	0	1	0	1
22	ZINC000014767590	0	4	0	0	3	1
23	ZINC000034944434	2	4	1	0	2	0
24	ZINC000002526388	2	4	1	1	0	1
25	ZINC000001090002	3	4	0	0	0	0
26	ZINC000008689960	3	1	0	0	0	0
27	ZINC000002097863	3	4	0	1	3	0
28	ZINC000005766341	1	4	0	0	3	1
29	ZINC000005811273	1	4	0	0	3	1
30	ZINC000006845904	0	4	1	0	3	1
	MIM1	2	2	0	1	0	1

BBB, blood-brain barrier; CYP2D6, cytochrome P-450 2D6; PPB, plasma protein binding.

Aqueous-solubility level: 0, extremely low; 1, very low, but possible; 2, low; 3, good.

BBB, level: 0, very high penetrant; 1, high; 2, medium; 3, low; 4, undefined.

CYP2D6 level: 0, noninhibitor; 1, inhibitor.

Hepatotoxicity: 0, nontoxic; 1, toxic.

Human-intestinal absorption level: 0, good; 1, moderate; 2, poor; 3, very poor.

PPB: 0, absorbent weak; 1, absorbent strong.

**TABLE 3:** Toxicities of compounds.

Number	Compounds	Mouse NTP		Rat NTP		Ames	DTP
		Female	Male	Female	Male		
1	ZINC000002572533	0	1	1	0.051	0.238	1
2	ZINC000040976869	0	1	1	0	0.021	0
3	ZINC000014883350	1	0	0	0.968	0	0
4	ZINC000100822245	0	1	1	0.051	0.238	1
5	ZINC000004096910	0	1	1	0	1	1
6	ZINC000230075702	0	0	1	0.02	0	1
7	ZINC000017044426	0	1	1	0.051	0.238	1
8	ZINC000004095521	0	1	1	0	0.017	0
9	ZINC000053147179	1	1	1	0	0	1
10	ZINC000003951623	0	0.999	1	0.023	0	0.824
11	ZINC000014951634	0.089	0	1	0	0	1
12	ZINC000049784088	0.995	0	0	0.008	1	1
13	ZINC000014883354	1	0	0	0.968	0	0
14	ZINC000013374322	0.002	0	1	0.015	0	0.095
15	ZINC000073280937	0.312	1	0	0.185	0	1
16	ZINC000014883346	1	0	0	0.968	0	0
17	ZINC000001577210	0	0.173	0	0.952	0	0.04
18	ZINC000019899011	0	1	0	1	0.802	0.999
19	ZINC000008552019	0	1	1	0.05	0.265	1
20	ZINC000038143593	0.061	0	0.274	0.088	0	1
21	ZINC000004104845	0.011	0.006	0.102	0.989	0	1
22	ZINC000014767590	0.494	0	0	1	0	0
23	ZINC000034944434	0	0	1	0.02	0	1
24	ZINC000002526388	0.999	0.041	0	0.999	0.999	0.745
25	ZINC000001090002	1	1	0	1	0.005	1
26	ZINC000008689960	0	0	0	0	0	0
27	ZINC000002097863	1	1	0	0	1	0
28	ZINC000005766341	0.917	1	1	0.006	1	0
29	ZINC000005811273	0.917	1	1	0.006	1	0
30	ZINC000006845904	0	1	1	0	0.014	0
	MIM1	0	0	0.163	1	0	1

NTP, U.S., national toxicology program; DTP, developmental toxicity potential.

NTP <0.3 (noncarcinogen); > 0.8 (carcinogen).

Ames <0.3 (nonmutagen); > 0.8 (mutagen).

DTP <0.3 (nontoxic); > 0.8 (toxic).

### 3.6 Ligand binding analysis

ZINC000013374322 and ZINC000001090002 were accurately connected to the binding pocket of MCL-1, and the mechanism of ligand binding was examined by using CDOCKER module. As shown in Table 4, the interaction energies of ZINC000001577210 (−40.7616 kcal/mol) and ZINC000001090002 (−43.3771 kcal/mol) are both much lower than MIM1 (−35.0968 kcal/mol), indicating that they may have higher binding affinity to MCL-1.

**TABLE 4:** DOCKER potential energy of compounds with MIM1, ZINC000013374322 and ZINC000001090002.

Compounds	-CDOCKER energy (Kcal/mol)	-CDOCKER interaction energy (Kcal/mol)
ZINC000013374322	44.039	40.7616
ZINC000001090002	31.916	43.3771
MIM1	5.95782	35.0968

**FIGURE 6:**
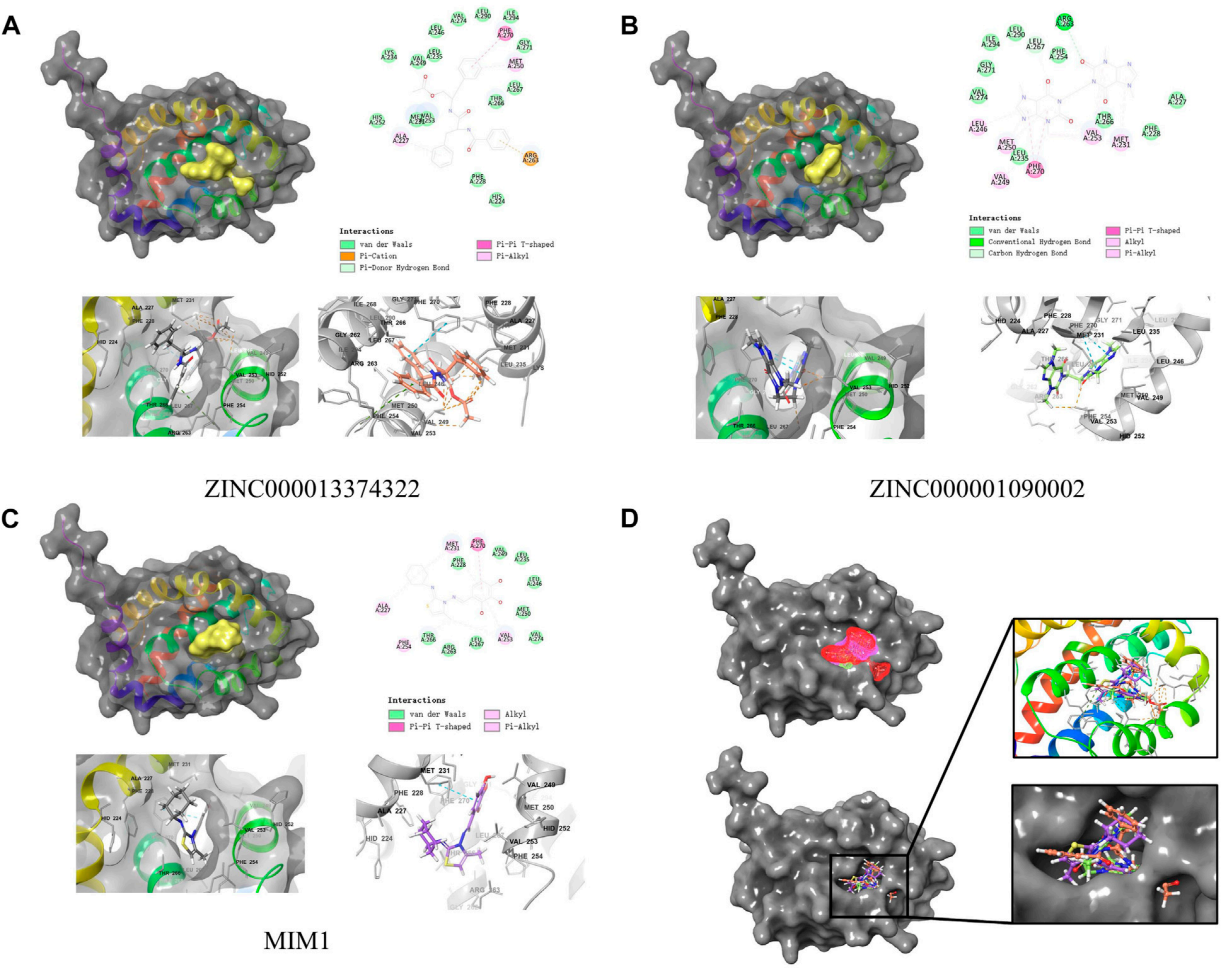
Schematic drawing of interactions between ligands and MCL-1 by Schrodinger and schematic of intermolecular interaction of the predicted binding modes. **(A)** ZINC000013374322-MCL-1 complex. **(B)** ZINC000001090002-MCL-1 complex. **(C)** MIM1-MCL-1 complex. **(D)** A comparison of the spatial conformation of small molecules in protein binding pockets and the gray surface of MCL-1 was added.

By applying DS2019 and Schrodinger software, we thoroughly analyzed the ligand conformation in the MCL-1 binding pocket and the protein-ligand complex interaction **(** Figures 6A–C). In these figures, the binding pattern of the two molecules to the MCL-1 binding pocket is visually shown. As shown in Figure 6D, there is a significant overlap between the two molecules and MIM1 in the binding pocket posture. According to Figure 6; Table 5, these two molecules and MIM1 are essentially identical in the way they bind and interact with MCL-1, proving that they have similar inhibitory effects on MCL-1. At the same time, it can be inferred that the two amino acid residues of PHE270 and MET250 play a crucial role in the functional domain of MCL-1.

**TABLE 5:** Hydrogen bond interaction parameters for each compound with MCL-1 residues.

Receptor	Compound	Donor atom	Receptor atom	Distances (Å)
MCL-1	ZINC000013374322	ARG263:HE	ZINC000013374322	3.01
		ARG263:NH1	ZINC000013374322	4.06
	ZINC000001090002	LEU267:HA	ZINC000001090002:O14	2.59
		ARG263:HH11	ZINC000001090002:O26	3.00
	MIM1	MIM1:H43	A:ALA227:O	1.96

Through the precise analysis of DS 2019, we showed detailed information on the interaction between ligand and protein, including bond length, bond type, bond atoms, and so on (Tables 5, 6). The results showed that ZINC000013374322 and ZINC000001090002 and MIM1 formed 2, 2, 1 pair of hydrogen bonds with MCL-1, respectively. In addition, ZINC000013374322 and ZINC000001090002, and MIM1 formed 12, 5, 8 pairs of hydrophobic interaction with MCL-1, respectively. Among them, although the hydrophobic interaction formed by ZINC000013374322 and MCL-1 are few, the bond lengths are small. So ZINC000013374322 forms a more stable hydrophobic interaction bonds with MCL-1. In conclusion, these results indicate that ZINC000013374322 and ZINC000001090002 may have better binding affinity to MCL-1 than MIM1, indicating that these two compounds have broad application prospects.

**TABLE 6:** Hydrophobic interaction related interaction parameters for each compound with MCL-1 residues.

Receptor	Compound	Donor atom	Receptor atom	Distances (Å)
6UDV	ZINC000013374322	ZINC000013374322	MET250	3.98
		PHE270	ZINC000013374322	4.9
		ZINC000013374322	ALA227	3.94
		ZINC000013374322	ZINC000013374322	5.65
		ZINC000013374322	ARG263	5.19
	ZINC000001090002	ZINC000001090002	VAL253	5.38
		ZINC000001090002	MET231	5.08
		PHE270	ZINC000001090002	4.86
		ZINC000001090002:C22	MET231	3.98
		ZINC000001090002	PHE270	4.6
		ZINC000001090002	LEU246	4.8
		ZINC000001090002:C22	VAL249	4.47
		ZINC000001090002	MET250	4.6
		ZINC000001090002	MET250	5.02
		ZINC000001090002:C22	VAL253	4.47
		ZINC000001090002	VAL253	5.14
		ZINC000001090002:C28	VAL253	4.86
	MIM1	MIM1:C1	ARG263	4.44
		MIM1	ARG263	5.49
		MIM1	VAL253	5.33
		MET250	MIM1	5.39
		PHE270	MIM1	5.05
		MIM1	MET231	4.66
		MIM1	ALA227	4.74
		VAL253	MIM1	4.96

### 3.7 Pharmacophore analysis and molecular dynamic simulation

**FIGURE 7:**
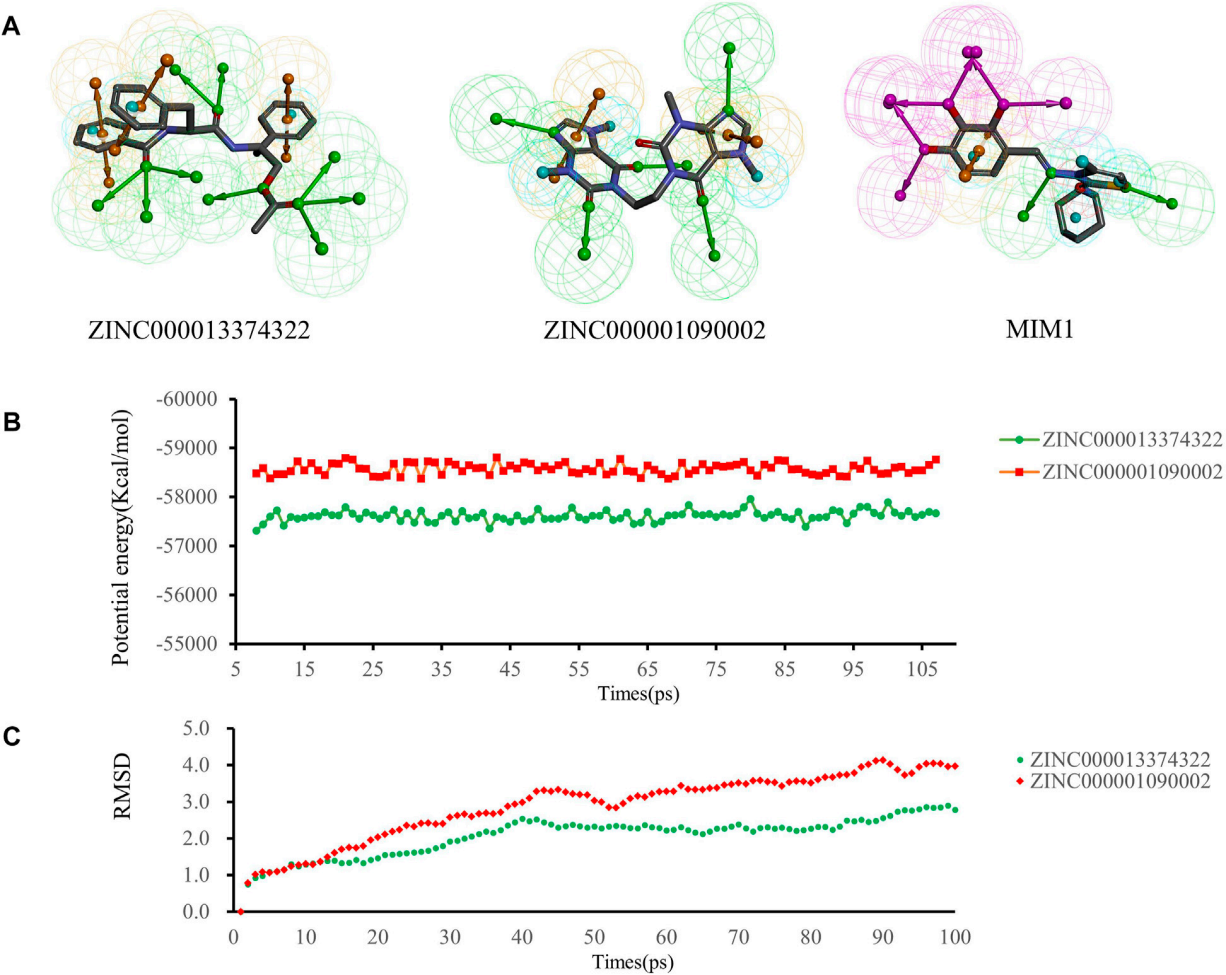
**(A)** Pharmacophore predictions using the 3D-QSAR module of DS2019 Green represents the hydrogen acceptor; blue represents the hydrophobic center; purple represents the hydrogen donor; yellow represents Aromatic Ring. **(B,C)** Results of molecular dynamics simulation of the compounds ZINC000013374322 and ZINC000001090002. **(B)** Potential energy, RMSD, root-mean-square deviation. **(B)** Average backbone root-mean-square deviation.

We performed pharmacophore analysis on these three molecules (Figure 7A). 9 and 5 hydrogen bond acceptors are displayed on the ZINC000013374322 and ZINC000001090002 respectively. There are 3 hydrophobic centers and 6 aromatic nuclei in ZINC000013374322 meanwhile ZINC000001090002 formed 3 hydrophobic centers and 4 aromatic nuclei. In summary, ZINC000013374322 has 18 characteristic pharmacophores and ZINC000001090002 has 12 characteristic pharmacophores. The characteristic pharmacophore of both molecules is basically the same as that of MIM1.

Molecular dynamics simulations were performed in simulated natural environments to evaluate the stability of ZINC000013374322-MCL-1 and ZINC000001090002-MCL-1complexes. As shown in Figures 7B, C, the potential energy and RMSD of each compound became stable over time, and the trajectory of the complex basically reached equilibrium after 15 ps. Molecular dynamics simulation results show that the interaction between these compounds and MCL-1 is beneficial to the stability of the complex. In conclusion, ZINC000013374322-MCL-1 and ZINC000001090002-MCL-1 complexes can stably exist in a natural environment and inhibit MCL-1 activity.

## 4 Discussion

Glioblastoma is a common brain tumor with a high degree of malignancy. Most patients have a poor prognosis and a very short survival time. The current conventional treatment methods for brain tumors have no significant improvement in the survival time and quality of life of glioblastoma. The occurrence and development of most tumors, including glioblastoma, are closely related to cell apoptosis. At present, a large number of studies have proved that the induction of apoptosis through a variety of ways can inhibit the progression of glioblastoma, which is the key way of drug treatment for glioblastoma. MCL-1 is a widely studied and potent anti-apoptotic protein that regulates cells by various mechanisms, including interactions with cell cycle regulators to affect cell division, acting as a molecular switch for double-strand break (DSB) DNA repair, regulation of autophagy and mitophagy through BH3-like protein interactions, etc. (Widden and Placzek, 2021). Down-regulating the expression and function of MCL-1 in tumor cells can effectively promote the apoptosis of tumor cells. Among them, the widely studied MCL-1 inhibitor is BH3-mimic, which has good effects on blood system tumors and multiple myeloma and has made great progress in combination with established therapies (Caenepeel et al., 2018). Among them, the widely studied MCL-1 inhibitors are BH3-mimics, including AZD5991, S63845, MIM1, etc. (Tron et al., 2018; Mallick et al., 2019; Moujalled et al., 2019; Wei et al., 2020), which have good effects on hematological tumors and multiple myeloma and have made great progress in combination with established therapies (Caenepeel et al., 2018). In recent years, targeted therapy has been widely used in the treatment of a variety of tumors and has achieved good results. By matching with genetic testing, targeted agents have significantly improved patient outcomes. However, GBM shows strong drug resistance, and the use of targeted drugs is severely limited [21] due to the permeability of the blood-brain barrier that limits drug delivery, low mutation burden, and suppression of the immune microenvironment (Yuan et al., 2022). However, the relatively mature small molecule inhibitors of MCL-1 that have been studied and developed are largely not used in the treatment of glioblastomas, probably due to difficulties in crossing the blood-brain barrier. Therefore, it is essential to study the role of MCL-1 in the prognosis prediction of glioblastoma patients and to develop safer and more effective MCL-1 inhibitors for the treatment of glioblastoma.

The present study aimed to establish an MCL-1-based prognostic model and to screen safe and effective MCL-1 inhibitors. Firstly, GBM samples were divided into two groups according to the expression level of MCL-1, and the immune signal enrichment level and differential gene expression were analyzed. Functional enrichment analysis of differential genes showed that these genes were in the extracellular matrix organization, and the expression level of MCL1_H group is significantly higher than that of MCL1_L group. NABA CORE MATRISOME and other aspects were enriched. Next, the prognostic MPS model was constructed and validated, which had high accuracy and sensitivity in predicting 1-year and 3-year survival rates of patients. By LASSO cox regression analysis, 5 genes were found to be independent prognostic factors: TSHR, HIST3H2A, AREG, OSMR, ARHGEF25. Glioblastoma highly expresses TSHR and TSH in the tumor microenvironment promotes its proliferation, invasion and immune evasion, which limits the T cell killing of glioblastoma. Treatment targeting intracranial TSH may reverse the immunosuppressive state of glioblastoma (Wu et al., 2022). miR-516a-5p downregulates the expression of HIST3H2A, thereby reversing the anti-proliferation effect induced by miR-516a-5p in NSCLC cells. miR-516a-5p may inhibit the proliferation of NSCLC cells by targeting HIST3H2A (Ye et al., 2019). AREG, one of the seven ligands that bind and activate EGFR, can promote the differentiation of T cells into Tregs in the tumor microenvironment, and targeting AREG in the tumor microenvironment may inhibit tumor invasion and immunosuppression (Coniglio and Segall, 2021). OSMR is a cell surface receptor that is a key component of EGFRvIII-STAT3 signaling, which forms a feedforward signaling mechanism with these molecules to drive glioblastoma genesis and progression (Jahani-Asl et al., 2016). ARHGEF25 promotes tumor cell migration, and serum-induced ARHGEF25 activation plays a key role in chemotactic migration by restricting lamellipodia formation to the direction of cell movement and keeping it at the leading edge (Hayashi et al., 2013).

Next, we chose the crystal structure of MCL-1 (PDB ID: 6UDV) and used MIM1 as the positive reference drug for the entire study. We first used the LibDock module of DS2019 to analyze the LibDock scores of compounds downloaded from the ZINC15 database. The 30 compounds with the highest LibDock scores were selected to analyze their pharmacological and toxicological properties using ADME and TOPKAT modules. In this step, several pharmacological properties of ZINC000013374322 and ZINC000001090002 were found to be superior to MIM1 and less toxic, so these two compounds were selected for further analysis. Next, the binding modes of the compounds and proteins were precisely analyzed using the CDOCKER module, and the results showed that the complex formed by these two compounds with MCL-1 had lower interaction energy, proving that their binding was more stable. Two amino acid residues, PHE270 and MET250 of MCL-1, were found to interact with two compounds and MIM1, indicating that these two amino acid residues were the key sites for inhibiting MCL-1 protein. This is the latest discovery in our study. At present, the specific functions of these two amino acid residues in MCL-1 have not been mentioned in other existing studies. We consider PHE270 and MET250 as the key amino acid residues in the MCL-1 pocket, and through modification and modification of them, the small molecule inhibitor can be more stably bound to the corresponding domain of MCL-1. In the development of more MCL-1 inhibitors, PHE270 and MET250 can be used as effective binding sites to select more reasonable inhibitors. We then analyzed the pharmacophores of the compounds and found that the compounds had similar characteristic pharmacophores. Finally, molecular dynamics simulations were performed for both compounds. In the simulated natural environment, the complex trajectories reached equilibrium after 30 ps, and the potential energy and RMSD of each complex tend to stabilize with time. Based on these results, ZINC000013374322 and ZINC000001090002 can be further modified to make the ligand binding to MCL-1 more reasonable and stable. Furthermore, few studies have been conducted on these two compounds, ZINC000013374322 (Aurantiamide Acetate) and ZINC000001090002 (Bisdionin B), and studies that have shown no effect on cancer, especially in the treatment of glioblastoma by inhibiting MCL-1 function. However, our study did demonstrate that they can effectively inhibit the MCL-1 function, which provides more prodrugs for the treatment of glioblastoma. Through further modification and improvement, these two compounds show excellent development prospects as MCL-1 small molecule inhibitors.

We have to admit that despite accurate measurements and virtual calculations in this study, there are still some limitations. Since there are many changes in the metabolism and transformation of drugs *in vivo*, corresponding experiments will be carried out in the future to verify other safety indexes of these two compounds, such as IC50, AB (aerobic biodegradability) and MTD (maximum tolerated dose), etc., to continuously optimize the structure of compounds and develop more reasonable drugs.

## 5 Conclusion

This study demonstrated that MCL-1 was a key factor affecting the prognosis of glioblastoma patients, and inhibition of MCL-1 can improve the prognosis of glioblastoma patients. We used lasso cox regression analysis to construct an MCL-1 related prognostic evaluation model and prognostic-related nomogram to predict the survival rate of glioblastoma patients. In addition, we found that TSHR, HIST3H2A, AREG, OSMR, and ARHGEF25 are novel independent factors affecting the diagnosis, treatment, and prognosis of GBM. Based on the role of MCL-1 in glioblastoma development, we used a series of computer-aided techniques to screen safer and more effective MCL-1 small molecule inhibitors from the ZINC15 database. ZINC000013374322 and ZINC000001090002 are safe and ideal drug candidates. In addition, this study also provided 30 candidate drugs and their pharmacological properties, which provided a new idea for the development and study of MCL-1 inhibitors.

## Data Availability

The original contributions presented in the study are included in the article/supplementary material, further inquiries can be directed to the corresponding author.
